# Boosting the cellular potency of embryonic stem cells by spliceosome targeting

**DOI:** 10.1038/s41392-021-00743-9

**Published:** 2021-08-31

**Authors:** Wilfried A. Kues

**Affiliations:** Friedrich-Loeffler-Institut, Federal Research Institute for Animal Health, Biotechnology, Stem Cell Physiology, Neustadt, Germany

**Keywords:** Embryonic stem cells, Embryonic stem cells

In recent work published in *Cell*, Shen et al.^1^ identified spliceosome inhibition in embryonic stem (ES) cells as a key mechanism for the transition from pluri- to totipotency. Spliceosome inhibition, achieved by RNA interference or the chemical inhibitor pladienolide B, may gain widespread relevance to the culture of totipotent ES cells, in vitro differentiation of extra-embryonal tissue and organoids, translation to the maintenance of pluripotent cells of other mammal species, including humans, and a better molecular understanding of cellular potency in stem cells and cancer.

The first successful isolation and maintenance of ES derived from the inner cell mass (ICM) of murine blastocyst stages was described in 1981,^2^ and since then acted as game changer for genetic studies in this mammalian model organism. In terms of cellular potency, ontogenesis is considered a one-way street with a continuous loss of potency from the totipotent zygote (and very early blastomere stages) to pluripotent ICM and then to less potent cells (multi-, di- and unipotent cells). It is generally accepted that germline-competent ES cells are pluripotent, meaning they can contribute to all cells of the adult body, but not to extra-embryonal tissues, such as yolk sac, ectoplacental cone, and extra-embryonic ectoderm. Nevertheless, the high self-renewal capability of ES cells allows complex genetic modifications in vitro. For genetic modification of the mouse genome, the pluripotent status is the precondition for the generation of cell-chimeric animals in blastocyst complementation assays, and the participation of the modified ES cells in gametogenesis.

However, an in-depth understanding of the molecular mechanisms demarcating totipotency of early embryonic cells (zygote, 2-cell and 4-cell embryonic blastomeres) and pluripotency of ICM and ES cells derived thereof is still fragmentary. In recent years, a number of publications described different forms/subgroups of pluripotency, termed as ground state, naive, primed, 2 cell-like, and extended potency,^3–5^ which made the once simple definition rather obscure. A direct comparison between the differently named pluripotent subgroups is rather difficult, since the studies were done with various ES cells from a number of mouse lines, different markers, culture media, methodologies, and partly also with ES cells of different species, mainly mouse and human.

Here, Shen et al. started out with a transcriptome analysis of early embryonic stages; ICM and ES cells, a more detailed analysis pinpointed to a coordinated upregulation of genes representing subunits of the spliceosome complex starting from the 8-cell stage and being continued in ICM and ES cells. The author speculated whether the spliceosome complex may be involved in the transition from toti- to pluripotency; to test their hypothesis, they transfected ES cells with short interfering RNAs against different spliceosome transcripts (the spliceosome consisted of 5 core and several cofactor subunits, here 14 transcripts were targeted), respectively. Transient repression of 10 of the 14 splicing factors resulted in ES cells, which maintained the typical colony morphology, however, pluripotent marker genes—Oct4 (Pou5f1), Nanog, Sox2, Zfp42 and others—became down-regulated, at the same time marker genes of totipotency—particularly Zscan4s and MERVL—were up-regulated. Zscan4s (Zink finger and SCAN domain containing 4) is a transcription factor and MERVL (murine endogenous retrovirus L) an endogenous retrovirus with a usually restricted expression to 2-cell embryos. These results were verified by supplementing the culture medium with pladienolide B, a specific spliceosome inhibitor, and culturing the ES cells for up to 16 passages. For these tests unmodified, but also transgenic ES cell lines carrying pluripotency reporter constructs (Rex1-GFP, mCherry-pre-mir-290) were employed. Again, after spliceosome inhibition, the reporter genes were turned off within few passages The reversibility of the pladienolide B treatment was shown in Oct4-GFP ES cells. The authors named the spliceosome-inhibited cells totipotent blastocyst-like cells (TBLCs), morphologically they resembled ES cells, but showed a somewhat reduced proliferation.

A critical question now was the cellular potency of the spliceosome-inhibited ES cells. Therefore blastocyst complementation assays with permanently genetically labelled TBLCs were designed. Either a group of six or a single TBLC were injected into a host blastocyst, transferred to a surrogate animal and different post-implantation stages, respectively, were recovered to assess the fate of the transplanted cells. In short, the TBLC-derived cells could be spotted in embryonic and extra-embryonic tissues, specifically yolk sac and placenta; whereas experiments with control ES cells resulted in a confined contribution to the embryo. Single-cell transcriptome sequencing identified a total of 15 embryonic and 6 extra-embryonic tissues with TBLC contribution, indeed strongly supportive of a bi-directional developmental capacity. A detailed molecular analysis of the TBLCs revealed that the splicing of pluripotent gene transcripts was indeed suppressed, but not the processing of totipotent pre-mRNAs. The authors speculated that this may be due to the few and short introns found in totipotent genes.

Essential is the replication of the results, and the assessment of the general validity in ES cells from other mouse lines. The long-term consequences of spliceosome repression also need a more detailed analysis, formally the germline competence of the here obtained viable chimeric mice needs to be shown. This includes the influence of the inhibitor treatment on in vivo development, and potential effects on cell fusion events. The translation of the methodology to pluripotent cells from other mammals is also highly tempting, since with the exception of rat ES cells, no robust germline-competent ES cells have been achieved so far.

**Fig. 1:**
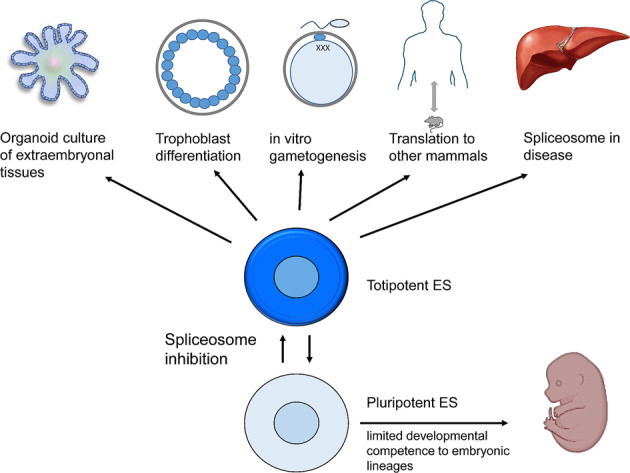
Potential applications of totipotent ES cells. Spliceosome targeting by RNA interference or the specific inhibitor pladienolide B seems to convert pluri- to totipotent ES cells, which were shown to have a bi-directional developmental capacity to embryonic and extra-embryonic cell lineages:^1^ allowing to explore in vitro differentiation of extra-embryonal tissue and organoids, translation to the maintenance of pluripotent cells of other mammal species, a better molecular understanding of cellular potency in stem cells and cancer, and potentially to develop more specific therapeutic treatments

The relevance of this study is the identification of the spliceosome as a key regulator of the transition between toti- and pluripotency. Specific spliceosome inhibitors, such as pladienolide B, may be instrumental for a detailed molecular understanding of the toti- to pluripotent transition, the in vitro development of extra-embryonal tissues and organoids, and the generation of artificial trophoblastoids (Fig. 1). The ability of TBLCs to generate artificial blastoids or entire fetuses remains to be solved. Potentially, the results may enable the establishment and maintenance of genuine ES cells in other mammals, including humans. An interesting aspect is whether the TBLC state will allow an efficient in vitro gametogenesis. In addition, the overlap of stem cell properties and cancer diseases may offer new therapeutic options.
